# Association between pro-inflammatory diet and ulcerative colitis: a systematic review and meta-analysis

**DOI:** 10.3389/fnut.2025.1586691

**Published:** 2025-06-18

**Authors:** Xianli Yin, Lv Tian, Qi Liu, Hanbing Zhao

**Affiliations:** ^1^The Clinical Medical College, Guizhou Medical University, Guiyang, China; ^2^Institute of Fundamental and Frontier Sciences, University of Electronic Science and Technology of China, Chengdu, China; ^3^Department of Gastroenterology, Affiliated Hospital of Guizhou Medical University, Guiyang, China

**Keywords:** pro-inflammatory diet, meta-analysis, ulcerative colitis, systematic review, PRIMSA

## Abstract

**Background:**

Emerging evidence suggests that dietary patterns can mediate intestinal inflammatory responses through immune-microbiome interactions. Diet and inflammation are important pathogenic factors for ulcerative colitis (UC). However, the existing evidence regarding the association between a pro-inflammatory diet and the risk of UC is controversial, and further clarification of this association is needed.

**Objective:**

This study aimed to explore the association between pro-inflammatory diet and UC risk.

**Methods:**

We systematically searched PubMed, Web of Science, Scopus, EMBASE, and Cochrane Library databases from their inception to January 15th, 2025. Two researchers independently used the Newcastle - Ottawa Scale to assess the quality of the included studies. Data analysis was performed using STATA 17 software.

**Results:**

This systematic review and meta-analysis included eight studies involving approximately 758,068 participants. The meta-analysis indicated that an inflammatory or pro-inflammatory diet did not increase the risk of UC (OR = 0.97, 95% CI = 0.84–1.12). However, subgroup analyses revealed differing results: within the case–control study subgroup, a pro-inflammatory diet was associated with an increased risk of UC (OR = 2.09, 95% CI: 1.23–3.56), whereas in the cohort study subgroup, no significant association was found between a pro-inflammatory diet and UC (OR = 0.91, 95% CI: 0.78–1.06). Sensitivity analysis indicated that the study results were robust. Additionally, Begg’s test (*p* = 0.174) and Egger’s test (*p* = 0.085) showed no significant publication bias in this study.

**Conclusion:**

The results of this study do not support a significant association between pro-inflammatory diets and UC risk. However, due to the limited level of evidence from observational studies and their heterogeneity, the association between pro-inflammatory diets and UC may be underestimated or overestimated. Therefore, larger multi-centre studies are needed to standardize the assessment of diets and adjust for microbial-related confounding factors in order to elucidate the association and mechanisms between pro-inflammatory diets and UC.

## 1 Introduction

Ulcerative colitis (UC) is a disease characterized by chronic gastrointestinal inflammation (1). It clinically presents with left lower quadrant abdominal pain, diarrhoea, weight loss, and rectal bleeding (2). It follows a chronic course marked by alternating relapses and remissions (3). Pathologically, UC is defined by superficial inflammation that initiates in the rectum and spreads continuously to the proximal colon, potentially involving the entire colon in severe cases (1, 4).

Over the past two decades, the global disease burden of UC has undergone substantial changes (5, 6). In most high-income countries, the prevalence has either stabilized or declined gradually. In contrast, it has increased rapidly in low-and middle-income countries, including those in Asia, Africa, and the Middle East. This disparity suggests that environmental factors, particularly dietary transitions, may drive the shift in disease epidemiology. The pathogenesis of UC involves complex interactions among genetic susceptibility, gut microbiota dysbiosis, and abnormal immune responses (7–9). For instance, genetic variants in genes such as NOD2 and IL-23R can increase the risk of the disease by affecting intestinal barrier function or immune cell activation, while gut microbiota dysregulation—manifested as imbalances in the Firmicutes/Bacteroidetes ratio and a reduction in short-chain fatty acid-producing bacteria—further exacerbates intestinal mucosal inflammation (10, 11). Environmental factors, including smoking, stress, and diet, modulate inflammatory responses via epigenetic or metabolic pathways, serving as critical disease triggers (7, 12–14). Piovani et al. also demonstrated that environmental factors, including dietary factors, are associated with the rising global prevalence of UC (15).

Dietary patterns play a crucial role in the occurrence and development of UC by modulating the gut microbiota, inflammation levels, and intestinal barrier function (16–18). For instance, the Western diet, characterized by high fat, high sugar, and low fibre content, may disrupt the intestinal barrier function, activate pro-inflammatory signalling pathways (e.g., NF - κB), and alter the composition of the gut microbiota, thereby exacerbating mucosal immune dysregulation (19, 20). Adherence to a Western dietary pattern, which is low in foods with anti-inflammatory and antioxidant properties, increases the risk of developing UC (21). Conversely, the Mediterranean diet, rich in polyphenols, *ω* - 3 fatty acids, and dietary fibre, may exert a protective effect on the gut by inhibiting inflammatory factors such as IL - 6 and TNF - *α* and regulating microbial metabolites like short-chain fatty acids (SCFAs) (22). A Mediterranean diet is associated with a reduced risk of UC (23). The relationship between pro-inflammatory diets and gut health has garnered increasing attention among various dietary patterns. The pro-inflammatory diet refers to a dietary pattern that may trigger or enhance inflammatory responses within the body through the intake of specific food components (24). Pro-inflammatory diets, typically high in saturated fatty acids and sugar and low in dietary fibre, alter the intestinal environment for microorganisms (25). Long-term consumption of such diets can lead to imbalances in gut microbiota, potentially resulting in compromised intestinal barrier function (26). In recent years, assessment tools for dietary inflammatory potential based on nutrients or food combinations have become a focal point of research. These include the Dietary Inflammatory Index (DII) (27), Empirical Dietary Inflammatory Pattern (EDIP) (28), and Inflammatory Score of Diet (ISD) (29, 30). By quantifying the pro-inflammatory or anti-inflammatory properties of diets, these tools offer a new perspective for exploring the association between diet and inflammatory diseases. Measures of dietary inflammatory potential have been applied in studies on ovarian cancer, colorectal cancer, and more (31, 32). Furthermore, a study in Iran reported a positive correlation between higher values of the Food-Based Dietary Inflammatory Index (FDII) and the risk of developing irritable bowel syndrome (IBS) (33).

As mentioned above, an pro-inflammatory diet may be associated with the occurrence and development of inflammatory bowel disease. However, a systematic review by Khademi et al. (34) indicates that current evidence regarding the association between pro-inflammatory diets and UC risk remains controversial. Studies by Shivappa et al. (35) and Khademi et al. (36) suggest that a pro-inflammatory increases the risk of UC. In contrast, other studies argue that there is no association between such diets and the development of UC (37, 38). Given the inconsistency of the research results and the close relevance of diet and UC to public health and clinical practice, we conducted a meta-analysis to comprehensively and systematically evaluate the existing evidence regarding the association between a pro-inflammatory and the risk of UC.

## 2 Methods

### 2.1 Registration information

This study adhered to the requirements of the Preferred Reporting Items for Systematic Review and Meta-Analyses guideline (39). Moreover, it was registered in the International Prospective Register of Systematic Reviews under the ID CRD420250652702.

### 2.2 Search strategy

We comprehensively searched for original studies on the association between pro-inflammatory (inflammatory) diet and ulcerative colitis in PubMed, Embase, Scopus, Cochrane Library, and Web of Science. The search period spanned from the inception of each database to January 15th, 2025. The search terms consisted of both subject headings and free-text words. The search strategy for PubMed was as follows: (((((((Inflammation [MeSH]) OR (Inflammation)) OR (Inflammatory)) OR (Inflammat*)) AND (diet [MeSH])) OR (pro-inflammatory diet)) OR (inflammatory diet)) AND ((ulcerative colitis [MeSH]) OR (ulcerative colitis)). This strategy was adapted for the other databases, with the terms adjusted according to each database’s syntax and indexing system.

### 2.3 Eligibility criteria

The inclusion criteria for the studies were as follows:

The study design should be a cohort or case–control study; The study must clearly define ‘pro-inflammatory diets’ and assess them using validated metrics that include but are not limited to (ISD, EDIP, and DII); The research content should focus on the association between a pro-inflammatory diet and the risk of UC; The study’s outcome measure should be the incidence of UC; The study should report the odds ratio (OR), relative risk (RR), or hazard ratio (HR) along with their 95% confidence intervals (CI) or provide sufficient data to calculate the effect size between a pro-inflammatory diet and UC; The literature should be published in English.

The exclusion criteria were as follows:

Duplicate literature; Review literature; Non-human studies, such as *in vitro*, *in vivo*, or animal studies; Literature from which the target data cannot be obtained; Irrelevant studies; Non-English literature.

### 2.4 Study selection

The retrieval results from all databases were imported into Endnote X9 software for duplicate removal and literature management. To ensure the accuracy and objectivity of the data, two independent reviewers (XLY and LT) initially screened the titles and abstracts of the retrieved literature according to the pre-set inclusion criteria. For the studies that initially met the criteria, the full-text articles were obtained and further screened to determine the final studies to be included. In case of disagreements between the two reviewers during the screening process, a consensus would be reached through discussion. A third reviewer (HBZ) would participate in the discussion and provide an arbitrating opinion if necessary.

### 2.5 Data extraction

This study strictly adhered to the PRISMA statement for data extraction to ensure the systematic nature of the research methodology. Two reviewers (XLY and LT) independently extracted data using a pre-tested data extraction form, and the third author (HBZ) cross-checked the accuracy of the results. The extracted data included the author (year), country, study design, age, gender (male/female), incident cases of UC, odds ratio (OR) with 95% confidence interval (CI), hazard ratio (HR) with 95% CI, assessment method, follow-up time (in years), quality (scores), and adjustment factors.

### 2.6 Quality assessment

The Newcastle-Ottawa Quality Assessment Scale is a quality assessment tool for non-randomized studies, which can be used to evaluate the quality of each study (40). It comprises eight items divided into three dimensions: cohort selection, comparability, and exposure/outcome. The maximum score of this checklist is 9 points. Studies with a score below four are considered low quality, those with a score of 4–6 are of medium quality, and those with a score of 7–9 are regarded as high-quality studies (40).

### 2.7 Data synthesis and analysis

To assess the association between a pro-inflammatory diet and ulcerative colitis, we pooled the odds ratios (ORs) and their corresponding 95% confidence intervals (CIs). Given the relatively low hazard ratio (HR) for UC and the expectation that the HR would yield estimates similar to those of the OR, all HRs were treated as ORs for the pooled analysis. First, the *Q*-test was employed to evaluate the heterogeneity among studies, with the significance level set at *p* = 0.1. Subsequently, the degree of heterogeneity was determined based on the *I*^2^ statistic: if *I*^2^ < 50%, indicating non - significant heterogeneity, a fixed-effects model was used; if *I*^2^ ≥ 50%, suggesting significant statistical heterogeneity (41), a random-effects model was selected. The following analyses were conducted: subgroup analysis according to the study design type; sensitivity analysis using the leave-one-out method (42); assessment of publication bias by observing the symmetry of the funnel plot and calculating the Begg’s test value and Egger’s test value (43, 44). The statistical software Stata 17.0 was used for data processing, and a *p*-value < 0.05 was considered statistically significant.

## 3 Results

### 3.1 Compliance with the registered protocol

There were no inconsistencies with the pre-registration protocol.

### 3.2 Study selection

**Figure 1:**
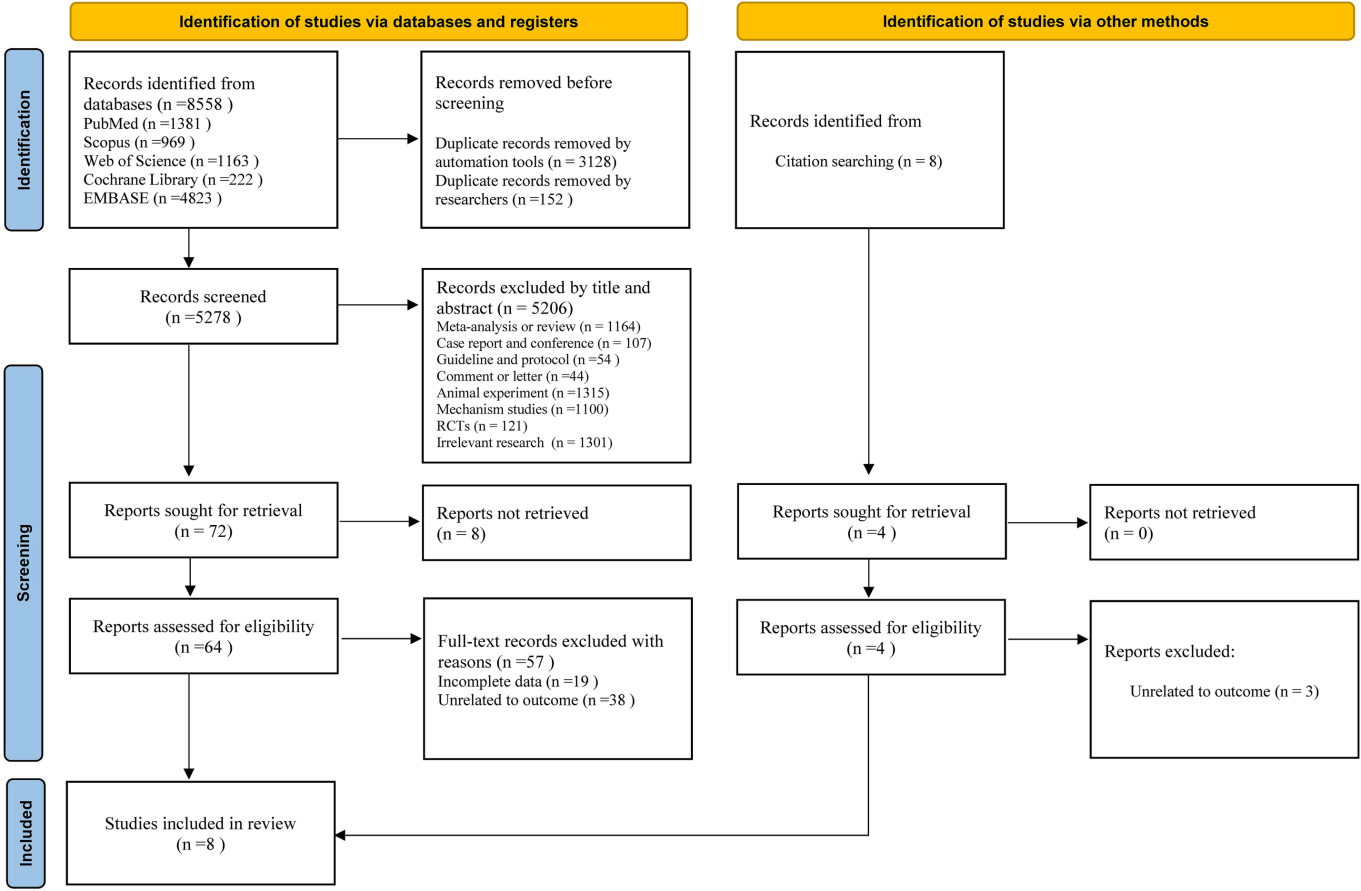
PRISMA flow chart for study selection.

The selection process and reasons for exclusion in this study are shown in Figure 1. We searched 8,558 documents in five databases: PubMed, Embase, Scopus, Cochrane Library, and Web of Science. After removing duplicates and filtering by title and abstract, we selected 72 studies for further evaluation. Eight studies were excluded because of the unavailability of full text, and the remaining 64 studies entered the full-text assessment stage. After full-text assessment, seven studies met the inclusion criteria. In addition, relevant citation tracking searches were conducted and supplemented. After applying the inclusion and exclusion criteria, eight studies (35–38, 45–48) were obtained for meta-analysis.

### 3.3 Study characteristics

This meta-analysis incorporated eight studies published between 2016 and 2025. The study types included prospective cohort studies and case–control studies. These investigations were carried out in various countries. Multiple prospective cohort studies covered several nations, such as European countries like Denmark, France, and Germany, as well as the United States, the United Kingdom, Spain, and Iran. The age range of the study participants was relatively broad, yet almost all participants were over 30 years old. Regarding gender, except for the study by Neeraj Narula, the other studies reported the numbers of male and female participants. The disease events were measured by the number of UC patients, and the number of UC patients in each study ranged from dozens to hundreds. When assessing the strength of the association between a pro-inflammatory diet and UC, the studies mainly used the OR and HR, along with their corresponding 95% CI. There was a diversity of methods for evaluating the relationship between diet and disease, including the Inflammatory Score of Diet (ISD), Empiric Dietary Inflammatory Pattern (EDIP), Dietary Inflammatory Index (DII), Food-based Dietary Inflammatory Potential (FDIP), and Inflammatory Potential of Diet (IPD). In terms of follow-up time, the follow-up period for the prospective cohort studies was 10.1–30 years, while the three case–control studies did not conduct follow-up. All the included studies were high-quality articles, with study quality scores of ≥7 points. All the included articles controlled for confounding factors, but the adjustment factors varied among studies (Supplementary Table S1). Common factors included smoking status, body mass index (BMI), physical activity level, energy intake, educational attainment, and alcohol consumption. Some studies also adjusted for factors such as hormone use and disease history according to their characteristics. Table 1 shows more information about each study’s main results.

**Table 1:** Characteristics of individual studies included in the meta-analysis.

Author (year)	Country	Study design	Age	Gender (Male/Female)	Incident (UC)	OR (95% CI)	HR (95% CI)	Assessment method*	Follow-up time (Year)	Quality (scores)	Adjustment factors
Meyer et al. (38)	Multiple countries1	Prospective cohort study	52.1 (SD 9.6)	125,656 / 268,599	459		0.85 (0.63–1.15)	ISD	13.6	High (8)	Adjusted 1
Lo et al. (37)	USA	Prospective cohort study	55 (IQR 29–85)	41,931 / 166,903	428		1.03 (0.78–1.36)	EDIP	NHS: 30 NHS II: 24 HPFS: 26	High (9)	Adjusted 2
Wellens et al. (48)	UK	Prospective cohort study	56.2	53,591 / 67,881	368		0.94 (0.65–1.35)	EDIP; DII	10.3	High (9)	Adjusted 3
Guevara et al. (45)	Spain	Prospective cohort study	48.9 (IQR 42.9 –56.0)	12,495/20,168	57		0.89 (0.63–1.26)	ISD	20.7	High (8)	Adjusted 4
Narula et al. (47)	2 Multiple countries	Prospective cohort study	35–70	Not mentioned	134		0.71 (0.41–1.24)	EDIP	10.1	High (7)	Adjusted 5
Khademi et a. (46)	Iran	Case-control study	Cases: 39.5; Controls: 41.5	108 / 219	109	1.12 (0.46–2.71)		FDIP	Not applicable	High (8)	Adjusted 6
Shivappa et al. (35)	Iran	Case-control study	Cases: 37.4±13.6; Controls: 36.2±11.9	85 / 105	62	2.58 (1.03–6.48)		DII	Not applicable	High (7)	Adjusted 7
Khademi et al. (36)	Iran	Case-control study	Cases: 39.5 ± 10.0; Controls: 41.5 ± 11.8	157 / 170	109	3.48 (1.32–9.10)		IPD	Not applicable	High (7)	Adjusted 8

UC, Ulcerative Colitis; OR, Odds Ratio; HR, Hazard Ratio; CI, Confidence Interval; SD, Standard Deviation; IQR, Interquartile Range; ISD, Inflammatory Score of the Diet; EDIP, Empirical Dietary Inflammatory Pattern; DII, Dietary Inflammatory Index; ISD, Inflammatory Score of the Diet; FDIP, Food-based dietary inflammatory potential; IPD, Inflammatory Potential of the Diet; NHS, Nurses’ Health Study; NHS II, Nurses’ Health Study II; HPFS, Health Professionals Follow-up Study.

Assessment method*: The Supplementary Table S2 presents definitions and differential characteristics for assessing dietary inflammation indices (ISD, EDIP, DII, FDIP, IPD).

Multiple countries 1: Denmark, France, Germany, Italy, the Netherlands, Norway, Spain, Sweden, UK.

Multiple countries 2: Argentina, Brazil, Canada, Chile, Poland, South Africa, Sweden.

Adjusted 1: Smoking status, BMI, physical activity, energy intake, educational level, alcohol intake.

Adjusted 2: Race, smoking, BMI, physical activity, use of non-steroidal anti-inflammatory drugs, use of oral contraceptives, use of hormone replacement therapy. Total fiber intake was additionally adjusted in some models during the analysis.

Adjusted 3: Age, sex, and ethnicity; socioeconomic factors like Townsend’s deprivation index (TDI) and education level; lifestyle factors including smoking status, drinking status, and physical activity; physical indicators such as body mass index (BMI) and total energy intake.

Adjusted 4: Age, sex, energy intake, smoking status, and physical activity.

Adjusted 5: Age, sex, household income, education, alcohol intake, smoking, energy intake, body mass index, waist-to-hip ratio, physical activity, anti-inflammatory drug use, oral contraceptive use, urban or rural location.

Adjusted 6: Age, sex, total energy intake, BMI, education, smoking status, diabetes history, physical activity, total fiber intake, regular meal pattern, chewing sufficiency, fluid consumption during a meal, fried food intake, fatty food intake.

Adjusted 7: Age, energy, sex, education, BMI, family history of IBD, appendectomy, smoking, H. pylori infection, non - steroidal anti - inflammatory drug (NSAID) use.

Adjusted 8: Age, sex, body mass index (BMI), education, smoking, medical history (diabetes), and physical activity.

### 3.4 Overall meta-analysis

**Figure 2:**
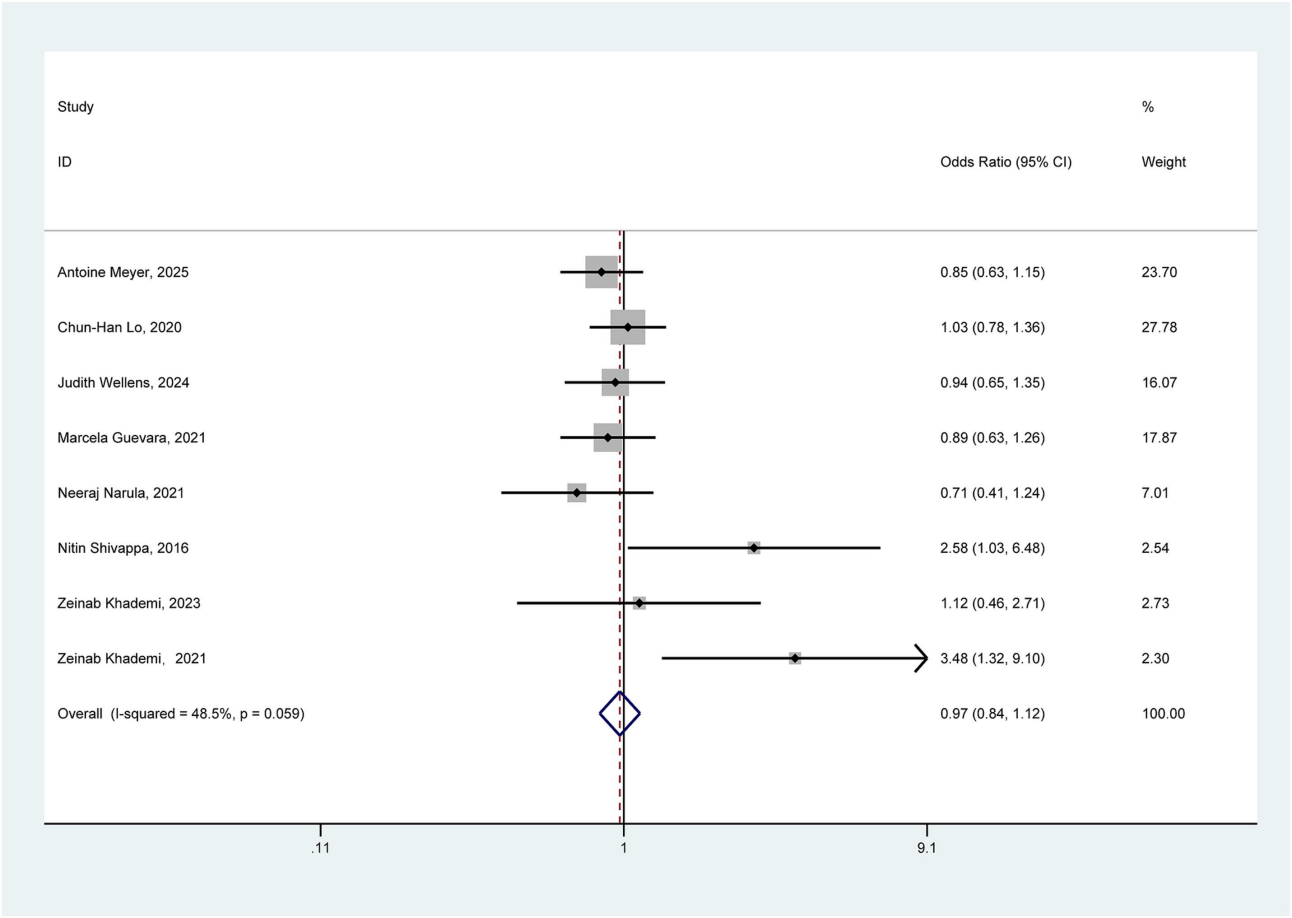
Forest plot of the association between pro-inflammatory diet and ulcerative colitis.

Eight studies exploring the association between pro-inflammatory diet and the risk of ulcerative colitis were incorporated into the comprehensive meta-analysis. Given the absence of significant heterogeneity (*I*^2^ = 48.5% < 50%, *p* = 0.059), Figure 2 presents the pooled outcomes derived from the fixed-effect model. The aggregated findings tentatively suggest that a pro-inflammatory diet may not significantly elevate the risk of UC (OR = 0.97, 95% CI: 0.84–1.12).

### 3.5 Subgroup analysis

**Figure 3:**
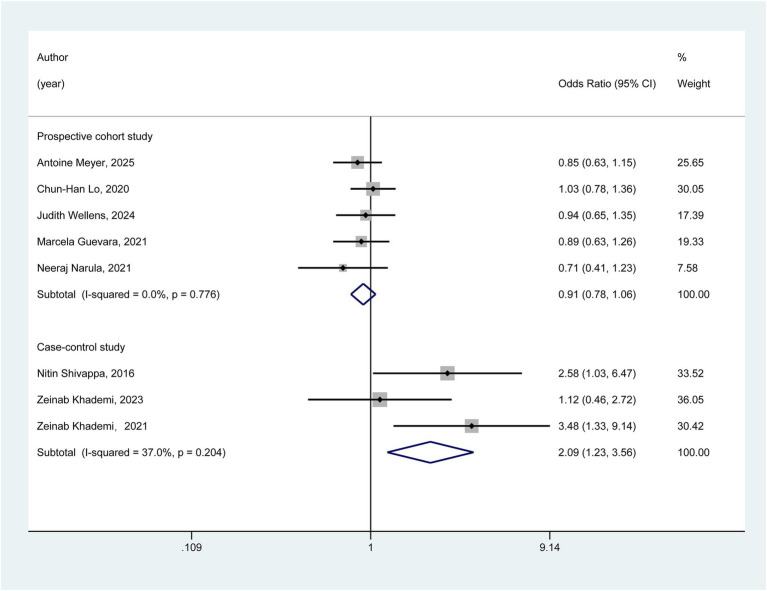
Subgroup analyses.

As depicted in Figure 3, we conducted subgroup analyses based on the study design. In the cohort studies subgroup, the pooled results indicated that no statistically significant association was observed between a pro-inflammatory diet and the risk of ulcerative colitis (OR = 0.91, 95% CI: 0.78–1.06). In contrast, within the case–control subgroup, the meta-analysis results suggested a potentially significant association between a pro-inflammatory diet and an increased risk of ulcerative colitis (OR = 2.09, 95% CI: 1.23–3.56).

### 3.6 Sensitivity analysis

**Figure 4:**
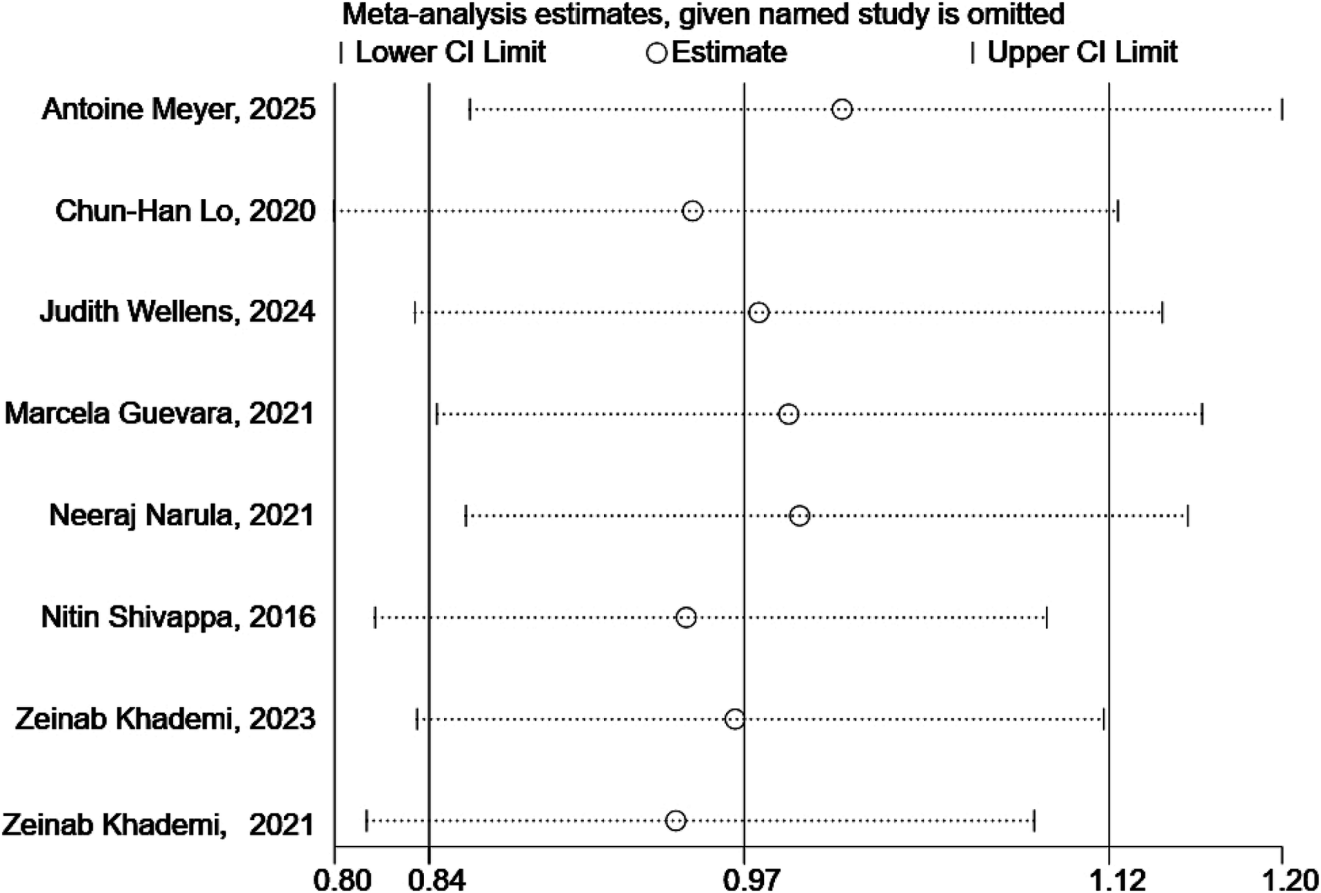
Sensitivity analyses.

As illustrated in Figure 4, the sensitivity analysis results demonstrated that the outcomes of the pooled analysis remained robust even after excluding any single study.

### 3.7 Publication bias

**Figure 5:**
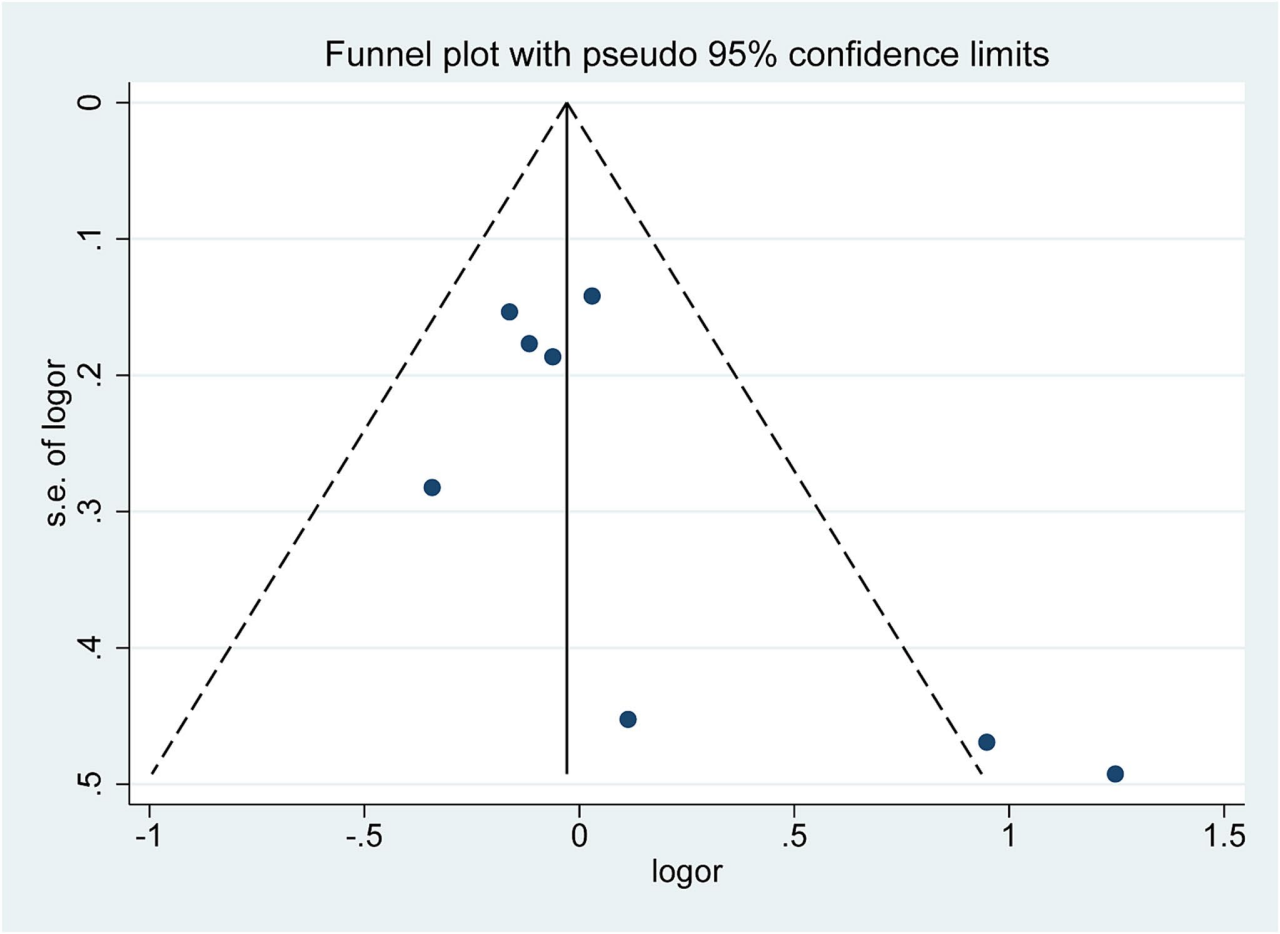
Funnel plot.

The funnel plot (Figure 5) exhibited a symmetrical distribution, significantly suggesting no publication bias in the meta-analysis results. Begg’s test (*Z* = 1.36, *p* = 0.174) and Egger’s test (*t* = 2.06, *p* = 0.085) findings further corroborated this conclusion (Supplementary Figure S1).

## 4 Discussion

Diet represents a complex set of exposure factors among common interactions. The cumulative effects of these factors can influence inflammatory responses and overall health. Several studies (49–52) have shown that diet can impact intestinal inflammation by altering the composition of the gut microbiota and the interplay between the gut microbiota and the local immune system. Previous research has explored the effects of different dietary patterns on inflammatory bowel disease (IBD). Racine et al. (53) reported that the Western dietary pattern is associated with an increased risk of IBD. In contrast, Khalili et al. (22) suggested that the Mediterranean dietary pattern may prevent the onset of IBD. However, the association between a pro-inflammatory diet and UC has been a subject of ongoing debate. Narula et al. (47) proposed no link between a pro-inflammatory diet and an increased risk of UC. Nevertheless, two case–control studies have observed that a pro-inflammatory diet significantly elevates the risk of UC.

Based on these inconsistent findings, this study conducted a meta-analysis of eight existing studies. The meta-analysis results tentatively indicated no significant association between a pro-inflammatory diet and the risk of developing UC. However, subgroup analyses based on different study designs yielded disparate outcomes. The subgroup of cohort studies showed no significant association, while the subgroup of the case–control studies suggested that a pro-inflammatory diet might increase the risk of UC. We believe this result is related to factors such as the study design type, the pro-inflammatory diet assessment method, and host biological characteristics (gut microbiota).

Regarding research design, cohort studies fall under the umbrella of prospective studies. By selecting study subjects and conducting long-term follow-up observations, these studies effectively control for confounding factors by systematically recording multiple characteristics of the subjects at the beginning of the study. However, during long-term follow-up, the dietary patterns of study subjects are susceptible to changes influenced by factors such as health education and variations in personal health status, which can reduce the accuracy of pro-inflammatory diet exposure assessment (54). Additionally, this research model requires a large sample size and extended periods, inevitably leading to sample attrition issues that can affect the reliability of research findings. Case–control studies, on the other hand, are retrospective studies that compare past pro-inflammatory diet exposure between case and control groups after the onset of disease, allowing for rapid research conclusions. Nevertheless, this method is prone to recall bias. Patients in the case group may have heightened awareness of their diet due to their illness, potentially reinforcing memories of a pro-inflammatory diet. In contrast, a lack of attention in the control group may lead to recall discrepancies. Such differences in recall between groups can distort the true association between exposure and disease (55).

In terms of assessment methods for pro-inflammatory diets, this study incorporated various evaluation systems, including the Inflammatory Diet Score (ISD) (29, 30), Empirical Dietary Inflammatory Pattern (EDIP) (28), Dietary Inflammation Index (DII) (27), Food-Based Dietary Inflammatory Potential (FDIP) (46), and Inflammatory Potential of Diet (IPD) (36). Due to differences in construction logic and measurement dimensions, various assessment methods have distinct criteria for evaluating pro-inflammatory diets. This heterogeneity directly affects the consistency and reliability of research results. Regarding assessment focus, different methods significantly differ in their attention to foods and nutrients. For instance, DII concentrates on key components such as antioxidants and fatty acids in food (56, 57), whereas EDIP emphasizes the overall effect of food combinations and nutritional pairings (58). Additionally, various assessment methods use different calculation formulas and weight distributions to quantify the inflammatory potential of diets, further increasing deviations in quantifying pro-inflammatory diets and interfering with the accuracy of the association between pro-inflammatory diets and the risk of UC (36, 59). Furthermore, significant differences exist in the feasibility and accuracy of assessment methods in practical applications (60). A combination of the above factors led to differences in the pro-inflammatory dietary assessment sessions across studies, affecting the results.

Regarding host biological characteristics (gut microbiota), the human intestinal flora can be divided into “enterotypes” dominated by Bacteroides or Prevotella. Different enterotypes exhibit varying inflammatory responses to the same diet (61). Cohort studies often recruit participants from community health screenings, and the *α*-diversity of the microbiota in community-based study populations is significantly higher than that of hospital-based study populations (62). High-diversity microbiota can buffer the harmful effects of pro-inflammatory diets through metabolic redundancy (63). For instance, in Bacteroides enterotype individuals, *Bacteroides thetaiotaomicron* can convert saturated fats into conjugated linoleic acid (CLA), which has anti-inflammatory properties (64). Conversely, case–control studies often include patients with a history of antibiotic use, and antibiotic-induced microbiota dysregulation can eliminate this protective effect (65). Animal experiments have shown that in antibiotic-treated mice, the incubation period for low-fiber diet-induced colitis is reduced by approximately 80% (66). Additionally, there is a critical time window effect in the microbiota-diet interaction, and dietary intervention during development is crucial for shaping the microbiota (67). Compared to cohort study populations, participants in case–control studies are relatively younger and have more plastic microbiota, which may amplify the negative effects of pro-inflammatory diets.

From the perspective of the potential mechanisms underlying the development of UC, intestinal microecological imbalance is one of the important risk factors for UC (7, 8). The high saturated fat, high sugar, and low dietary fiber content in a pro-inflammatory diet can change the living environment of gut microorganisms (25). Long-term consumption of such a diet leads to a decrease in the abundance of beneficial bacteria such as Bacteroides in the gut, an imbalance in the ratio of Firmicutes to Bacteroidetes, and potentially impairs the intestinal barrier function (26). Simultaneously, the metabolites of the gut microbiota also change, with a reduction in the production of SCFAs (68). SCFAs provide energy for intestinal epithelial cells, maintain their normal function, and possess anti-inflammatory properties. A decrease in their production weakens the gut’s anti-inflammatory capacity, making the intestinal mucosa more susceptible to inflammatory damage (68). Moreover, a pro-inflammatory diet may activate the host’s immune cells and affect the immune regulatory network (69). When excessive pro-inflammatory foods are consumed, immune cells such as T and B cells in the gut-associated lymphoid tissue are abnormally activated, releasing many pro-inflammatory cytokines, such as IL-17 and IFN - *γ* (70). These cytokines further recruit inflammatory cells, intensify the intestinal inflammatory response, and promote the development of UC (71).

Although our findings do not support a significant association between a pro-inflammatory diet and the risk of developing UC, this conclusion may be limited by various factors, including the assessment methods for inflammatory indices and the number of available studies, which may have reduced the level of evidence. Therefore, further large-scale, high-quality clinical studies and basic experiments are needed to elucidate the relationship between pro-inflammatory diets and UC systematically.

This meta-analysis has several limitations:

- Limited number of studies: The eight studies included are insufficient to comprehensively account for the impacts of diverse populations, geographical regions, and lifestyles worldwide on the relationship between a pro-inflammatory diet and UC. Substantial genetic variations among populations in different regions may influence individual metabolic and immune responses to a pro-inflammatory diet.
- Problems with dietary assessment methods: The eight included studies employed diverse approaches to assess the overall dietary inflammatory index, such as the Inflammatory Score of Diet (ISD), Empirically Derived Dietary Inflammatory Pattern (EDIP), and Dietary Inflammatory Index (DII). These assessment methods mostly rely on self-reported dietary information, each with its own focus, and are subject to measurement errors and limitations.
- Limitations in study types: Most studies did not conduct in-depth analyses of the associations between diet and the course of UC (e.g., disease activity and recurrence rate). As a result, it is impossible to determine the influence of specific factors on the outcomes. In addition, case–control studies and cohort studies are observational studies with relatively low levels of evidence; therefore, the quality of evidence derived from our findings is limited.

Despite the aforementioned limitations, this meta-analysis still boasts some significant advantages:

- The sensitivity analysis indicates that the research findings are robust and reliable. The results of Begg’s and Egger’s tests suggest the absence of publication bias, enhancing the credibility of our synthesis of the available evidence.
- This study represents the first meta-analysis to explore the relationship between a pro-inflammatory diet and the risk of ulcerative colitis. As such, it can offer valuable, evidence-based guidance for clinical practice, potentially informing preventive strategies related to ulcerative colitis.

Clinically, individuals with high pro-inflammatory dietary patterns may require attention to gut health monitoring based on their specific characteristics. Future research should deeply explore the underlying mechanisms (such as gut microbiota and genetic susceptibility) between a pro-inflammatory diet and UC through multi-omics studies and conduct interventional studies to verify the real impact of dietary patterns on UC, providing a basis for precise prevention.

## 5 Conclusion

In summary, this study does not support a significant association between pro-inflammatory diets and UC risk. However, subgroup analyses revealed that the type of study design may affect the interpretation of the results. Clarifying the relationship between pro-inflammatory diets and UC is of great public health significance for developing scientific and effective dietary intervention strategies to reduce the morbidity and disease burden of UC, and it is expected to open up new pathways for the prevention of UC. In the future, more large-scale and high-quality clinical studies and basic experiments are needed to elucidate the association between pro-inflammatory diets and UC systematically and to provide an evidence-based basis for personalized dietary interventions.

## Data Availability

The original contributions presented in the study are included in the article/Supplementary material, further inquiries can be directed to the corresponding author.
