# Supplementary material for: Association between pro-inflammatory diet and ulcerative colitis: a systematic review and meta-analysis
Source: Front Nutr. 2025 Jun 18;12:1586691. doi: 10.3389/fnut.2025.1586691 (PMC12213411; doi:10.3389/fnut.2025.1586691)

**Association between pro-inflammatory diet and ulcerative colitis: a systematic review and meta-analysis**

[Table S1. Adjustment factors of individual studies included in the meta-analysis. 2](#_Toc13059)

[Table S2. Assessment methods of individual studies included in the meta-analysis. 3](#_Toc10985)

[Figure S1 4](#_Toc27428)

# Table S1. Adjustment factors of individual studies included in the meta-analysis.

| **Author (year)** | **Demographic Characteristics** | **Lifestyle** | | | **Physiological Metrics - Anthropometry** | **Medical/Infection History** | **Medication**  **Use** | **Socioeconomic**  **Factors** | **Other Specific Factors** |
| --- | --- | --- | --- | --- | --- | --- | --- | --- | --- |
|  |  | **Tobacco & Alcohol** | **Diet & Nutrition** | **Physical Activity** |  |  |  |  |  |
| Antoine Meyer, 2025 | Educational level | Smoking status, alcohol intake | Energy intake | Physical activity | BMI | - | - | - | - |
| Chun-Han Lo, 2020 | Race | Smoking | Total fiber intake | Physical activity | BMI | - | Non-steroidal anti-inflammatory drugs,  oral contraceptives,  hormone replacement therapy | - | - |
| Judith Wellens, 2024 | Age, sex, ethnicity | Smoking status, drinking status | Total energy intake | Physical activity | BMI | - | - | Townsend’s deprivation index (TDI),  education level | - |
| Marcela Guevara, 2021 | Age, sex | Smoking status | Energy intake | Physical activity | - | - | - | - | - |
| Neeraj Narula, 2021 | Age, sex, urban or rural location | Smoking, alcohol intake | Energy intake | Physical activity | BMI,  waist-to-hip ratio | - | Anti-inflammatory drug use,  oral contraceptive use | Household income,  education | - |
| Zeinab Khademi, 2023 | Age, sex, education | Smoking status | Total energy intake,  total fiber intake,  regular meal pattern,  chewing sufficiency,  fluid consumption during a meal,  fried food intake,  fatty food intake | Physical activity | BMI | Diabetes history | - | - | - |
| Nitin Shivappa, 2016 | Age, sex, education | Smoking | Energy | - | BMI | Family history of IBD,  appendectomy,  H.pylori infection | Non-steroidal anti-inflammatory drug (NSAID) use | - | - |
| Zeinab Khademi，2021 | Education | Smoking | - | Physical activity | BMI | Medical history (diabetes) | - | - | - |

# Table S2. Assessment methods of individual studies included in the meta-analysis.

| **Assessment method** | **Full Name** | **Core Indicators** | **Assessment Dimensions** | **Data Sources** | **Difference Characteristics** |
| --- | --- | --- | --- | --- | --- |
| ISD | Inflammatory Score System | 12 pro-/anti-inflammatory nutrient ratios | Macronutrient balance | 24-hour dietary recall | Focuses on short-term dietary modulation; high sensitivity to micronutrients |
| EDIP | Empirical Dietary Inflammatory Pattern | 39 food group inflammatory scores | Synergistic effects of food combinations | FFQ dietary questionnaire | Uses principal component analysis (PCA) to capture biological interactions |
| DII | Dietary Inflammatory Index | 45 nutrient inflammatory weights | Nutrient-gene interactions | Standardized food databases | Cross-culturally validated; incorporates dose-response relationships |
| FDIP | Food-Driven Inflammatory Pattern | 28 processed food inflammatory coefficients | Food processing levels and additives | Market-sampled foods | Integrates NOVA classification; emphasizes ultra-processed foods |
| IPD | Inflammatory Potential of Diet | Personalized metabolic trajectories | Diet-metabolome-microbiome interactions | Multi-omics data | Machine learning-driven dynamic assessment for individualized predictions |

# Figure S1


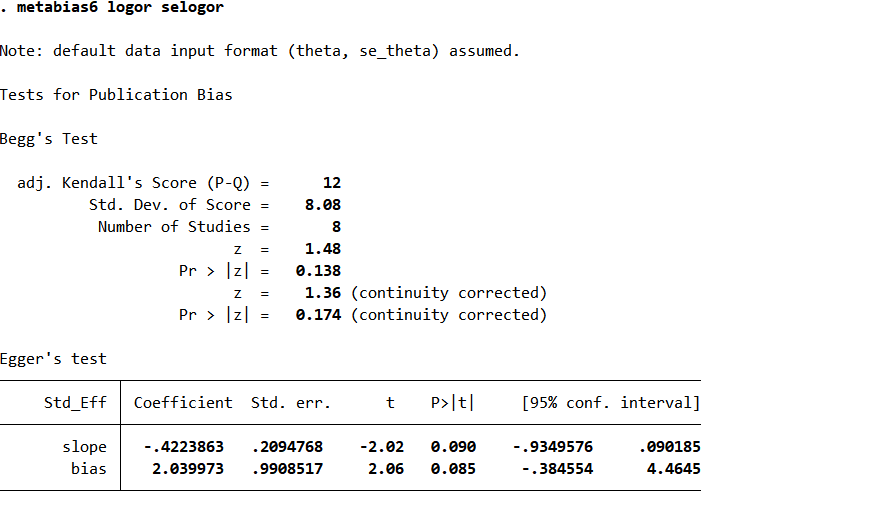

Supplement: Supplementary file 1 [file Supplementary_file_1.DOCX]
